# The effect and mechanism of YH0618 granule on chemotherapy- induced hair loss in patients with breast cancer: study protocol for a randomized, double-blind, multi-center clinical trial

**DOI:** 10.1186/s13063-019-3893-3

**Published:** 2019-12-12

**Authors:** Jie-shu You, Li Guo, Mei Huang, Xin-lei Shi, Man-di Lin, Zhen Guo, Ya-li Cao, You-zhi Sun, Qian Xu, Wei-ling Qu, Huan-lan Liu, Jian-ping Chen

**Affiliations:** 1 School of Basic Medical Sciences, Guangzhou University of Chinese Medicine, Guangzhou, Guangdong Province China; 2 School of Chinese Medicine, The University of Hong Kong, 10 Sassoon Road, Pokfulam, Hong Kong; 3 Galactophore Department, Guangdong Provincial Hospital of Chinese Medicine, Guangzhou, Guangdong Province China; 4 Galactophore Department, The First Affiliated Hospital of Guangzhou University of Chinese Medicine Guangzhou, Guangzhou, Guangdong Province China; 5 Galactophore Department, The Third Hospital of Nanchang, Nanchang, Jiangxi Province China; 6 Basic Medical College, Jiangxi University of Chinese Medicine, Nanchang, Jiangxi Province China; 7 Shenzhen Institute of Research and Innovation, The University of Hong Kong, Shenzhen, Guangdong Province China

**Keywords:** Medicinal and edible compound prescription, YH0618 granule, Chemotherapy-induced hair loss, Taxanes, Anthracyclines, Kidney deficiency and renal dysfunction, Quality of life

## Abstract

**Background:**

Hair loss is one of the most common side effects of chemotherapy, and can cause persistent negative emotions, further affecting therapeutic effects and reducing the quality of life. However, there are no clinically safe and effective methods to solve the problem at present. Our previous clinical and animal studies showed that a medicinal and edible decoction, YH0618, could significantly promote hair growth in cancer patients after chemotherapy, without interfering with the anti-tumor effects of chemotherapy. Besides, the theory of Chinese Medicine believes that the “Essence of the kidney is reflected on the hair”. Therefore, this study will further explore the efficacy of YH0618 granule on chemotherapy-induced hair loss in patients with breast cancer by a randomized, double-blind, multi-center clinical trial and elucidate the potential mechanism from the aspect of kidney deficiency or renal dysfunction.

**Methods/design:**

Eligible breast cancer patients who will start chemotherapy will be randomly divided into group A (YH0618 granule) and group B (placebo). The chemotherapeutic agents contain taxanes or/and anthracyclines, and the chemotherapy regimen will be for at least six cycles with a cycle every 3 weeks. Subjects assigned to group A will receive YH0618 granules twice a day (6 g each time), 6 days a week, mixed with 300 ml warm water from the first to the fourth chemotherapy cycle. Subjects in group B will receive the placebo granule in the same manner. The primary outcome is the time point of occurrence of hair loss reaching grade II as assessed by the WHO Toxicity Grading Scale, and objective indices of hair quality and hair-follicle growth recorded by a hair and scalp detector before the fifth chemotherapy cycle. Secondary outcomes include changes of facial color and thumbnail color, grading of thumbnails ridging, assessment of quality life, level of fatigue, routine blood test results, hepatic and renal function, and certain medical indicators which can reflect kidney deficiency in Chinese Medicine.

**Discussion:**

This research is of great significance for the treatment of cancer and improving the quality of life of cancer patients. The study may provide the most direct evidence for meeting clinical needs and lay a solid scientific foundation for later product development.

**Trial registration:**

Chinese Clinical Trial Registry, ID: ChiCTR1800020107. Registered on 14 December 2018.

## Background

Chemotherapy is a major type of cancer treatment using chemical medications to affect cancer cell growth, division and reproduction. Regardless of the route of administration, chemotherapy drugs are introduced into the blood stream, so that chemotherapy can cause various degree of damage to other normal organs and tissues while killing cancer cells, further causing a series of serious adverse effects/toxicity.

Hair loss is an obvious side effect of chemotherapy. The incidence of chemotherapy-induced hair loss is as high as 65% in patients receiving chemotherapy and in some is even up to 80–100% in patients receiving specific agents, such as doxorubicin and docetaxel [1–3]. Although hair loss itself does not cause damage to the body and threaten life, it can induce persistent negative emotions such as anxiety, depression and negative evaluation of self-image, which in turn reduces quality of life [4]. The hair loss caused by chemotherapy is usually reversible; however, in most cases, the color of the new hair is grayish or different from the previous color, and the hair texture also shows some changes, such as being rougher, slower growing and sparser [5, 6]. Besides, contemporary social media and excessive attention to appearance have put more pressure on patients, with 8% of patients saying that they refuse chemotherapy because of fear of alopecia [7]. Even some female patients said that having no hair is more difficult to tolerate than mastectomy [8].

The mechanism of chemotherapy-induced hair loss is still unclear because of the difference between animal models and the actual human body, and the human scalp cannot be extracted for research. The current reported mechanisms of chemotherapy-induced hair loss mainly involve deoxyribonucleic acid (DNA) damage, hair-follicle cell-cycle inhibition, hair-follicle-cell apoptosis, and reactive oxygen species and signal transduction, etc. Accordingly, animal-model studies have found that vasoconstrictors, antioxidants, hair-growth cycle regulators and parathyroid hormone can improve hair loss caused by chemotherapy [9]. In clinical practice, it has been reported that minoxidil, AS101 and vitamin D3 can treat chemotherapy-induced hair loss, but the effect is not significant [10]. Currently, scalp-cooling is the only method approved by the US Food and Drug Administration (FDA) for use for chemotherapy-induced hair loss, and the hypothesis about its mechanism is that the low-temperature-induced rapid contraction of blood vessels can reduce blood flow into hair follicles, and cause a general reduction in cutaneous-cell metabolism, which makes the hair less affected by the chemotherapy [11]. Unfortunately, the success rate of scalp-cooling is also only 50%, and patients with cold allergy, cold agglutination, and cold globulinemia are not suitable for this method [9]. Although some progress has been made in the mechanism, research and management of alopecia, there is no very effective way in solving the hair loss caused by chemotherapy so far. Therefore, it is necessary for clinicians and researchers to pay more attention to chemotherapy-induced alopecia and a series of relevant psychological problems to further elucidate the mechanism of hair loss and develop safe and effective solutions.

YH0618, a medicinal and edible compound prescription, is developed based on the “homology of medicine and food,” theory ancient prescription, and long clinical practice. Our previous animal studies have shown that YH0618 decoction did not interfere with the anti-tumor effects of chemotherapy drugs [12]. Additionally, a randomized clinical trial also showed that YH0618 significantly accelerated hair regrowth and reduced thumbnail pigmentation in cancer patients who have completed chemotherapy (data was not showed, but the protocol was published in [13]). Therefore, this study will further explore the efficacy of YH0618 granule on chemotherapy-induced hair loss in patients with breast cancer by a randomized, double-blind, multi-center clinical trial. YH0618 consists of five medicinal and edible foods (black soybean and liquorice, etc.) which are recommended by clinicians for cancer patients and all components have a history of safe use in other foods. Besides, each of the components possesses a distinct pharmacological profile, including removing free radicals in the body, regulating the immune system, preventing cancer, detoxifying and enhancing the sense of taste [14–16]. Black soybean and liquorice, as the main essential ingredients, have been used for detoxification for millennia in China. Based on the theory of the “Essence of the kidney is reflected on the hair” in traditional Chinese Medicine, the color, texture and growth of hair is believed to be closely related to the kidney. However, there is no in-depth research on the relationship between kidney and hair, and no research is combined with the comprehensive evaluation of renal function from the aspects of both Chinese and western medicine to explore the mechanism of chemotherapy-induced hair loss. The kidney is an important detoxification organ of the human body and helps to filter out toxins in the blood and other waste through urine. Chemotherapy drugs may not cause renal organic lesions, but they “consume” kidney essence and kidney *qi*, which breaks the balance of the body. Therefore, we believe that chemotherapy agents not only directly produce toxic effects on hair-follicle cells, but also deplete *qi*, blood and body fluids, especially kidney essence and kidney *qi*, and breaks the balance of *yin* and *yang* of the human body, which leads to the obstruction of microcirculation and the decline of immune function, further resulting in nutritional disorders of hair follicles and hair loss. Thus, the study will also elucidate the mechanism of YH0618 granule on reducing hair loss from the aspect of kidney deficiency or renal dysfunction. The hypothesis of the study is that YH0618 granules could delay chemotherapy-induced hair loss by improving kidney deficiency and renal dysfunction.

## Methods/design

### Study design

**Fig. 1:**
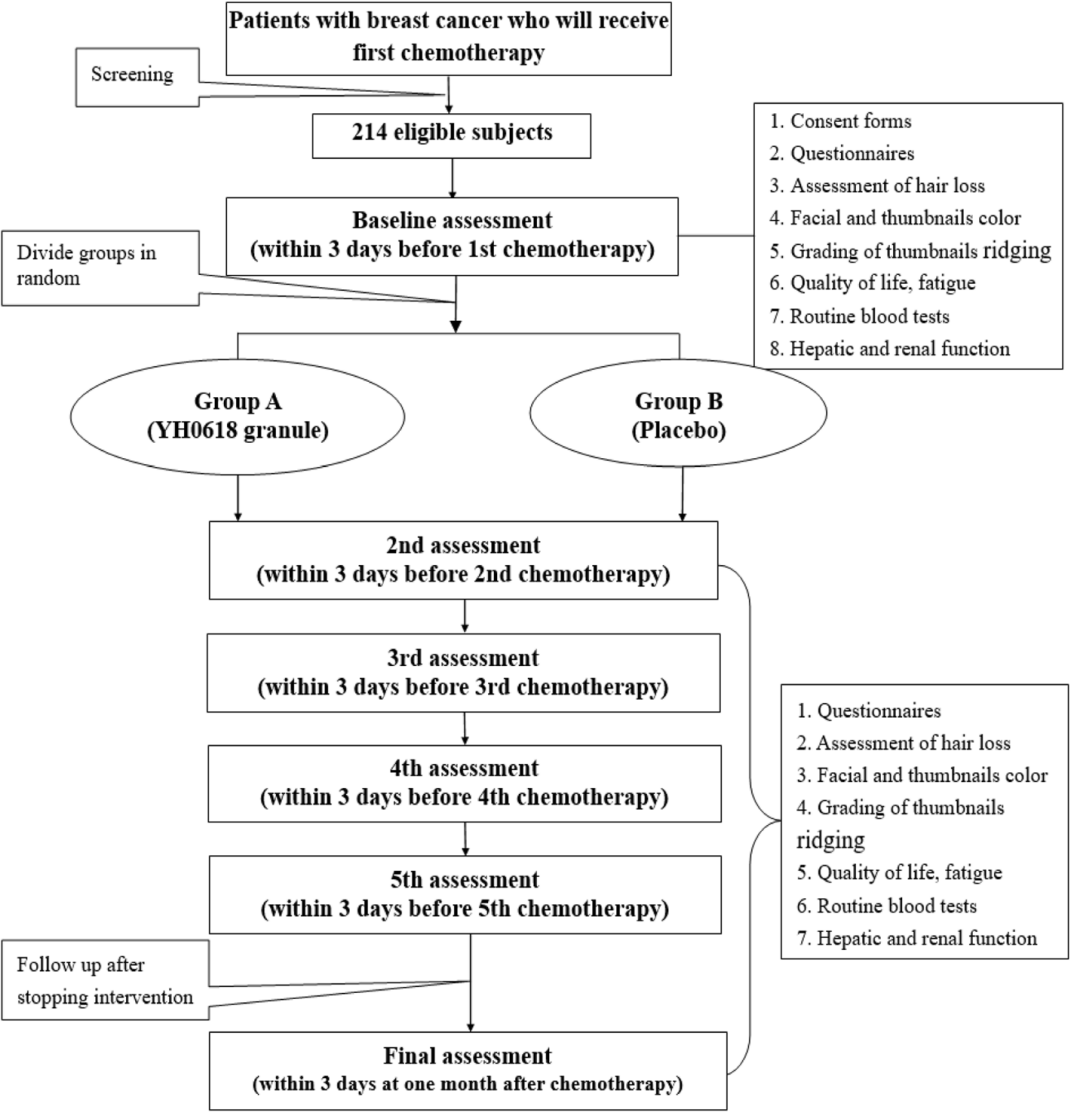
Flow chart of the clinical trial

This is a randomized, double-blind, multi-center controlled trial which aims at exploring the efficacy of YH0618 granule on chemotherapy-induced hair loss in patients with breast cancer and elucidating the potential mechanism from the aspect of kidney deficiency or renal dysfunction. To achieve this goal, a total of 214 breast cancer patients who will receive their first chemotherapy will be recruited for the study. The patients will be randomly divided into group A (YH0618 granule) and group B (placebo) using a 1:1 allocation ratio, adhering to the Consolidated Standards of Reporting Trials (CONSORT) Statement [17] and the Standard Protocol Items: Recommendations for Interventional Trials (SPIRIT) Statement [18] (Additional file 1). The primary outcome of this study is the time point of occurrence of hair loss reaching grade II as assessed by the World Health Organization (WHO) Toxicity Grading Scale, and objective indices of hair quality and hair-follicle growth recorded by a hair and scalp conditioner (CBS-603, CBS-Medical Skin Analysis, Taiwan). Secondary outcomes include changes of facial color and thumbnail color, grading of thumbnail ridging, assessment of quality life, fatigue level, routine blood test results, hepatic and renal function, and some medical indicators which can reflect kidney deficiency in Chinese Medicine. The flow chart of the study is shown in Fig. 1 and Table 1. The recruitment, evaluation and data collection will be conducted at Galactophore Department of Guangdong Provincial Hospital of Chinese Medicine, Galactophore Department of The First Affiliated Hospital of Guangzhou University of Chinese Medicine, and Galactophore Department of The Third Hospital of Nanchang.

**Table 1:** Trial process chart

	Before baseline screening	Baseline	Visit 1 treatment phase	Visit 2 treatment phase	Visit 3 treatment phase	Visit 4 treatment phase	Visit 5 follow-up phase
		(before chemo)	Before 2nd chemo	Before 3rd chemo	Before 4th chemo	Before 5th chemo	1 month after chemo
Patients							
Inclusion and exclusion criteria	×						
Informed consent		×					
Demographics		×					
Medical characteristics	×	×	×	×	×	×	×
Randomization and allocation concealment		×					
Primary outcomes							
Grading of hair loss		×	×	×	×	×	×
Objective indices (hair quality and hair follicles)		×	×	×	×	×	×
Secondary outcomes							
Facial color and thumbnail color		×	×	×	×	×	×
Grading of thumbnail ridging		×	×	×	×	×	×
Quality of life and fatigue		×	×	×	×	×	×
Routine blood test		×	×	×	×	×	×
Liver function (ALT, AST, total protein, and Alba)		×	×	×	×	×	×
Renal function (Cr, UA and BUN)		×	×	×	×	×	×
Kidney deficiency (IgM, C3、CD4 +, CD8 + Mg^2+^, Cu^2+^, Zn^2+^, Fe^3+^)		×		×		×	
Adverse events			×	×	×	×	×
Patients’ diary records			Every day during the study				

*BUN* blood urea nitrogen, *Cr* creatinine, *ALT* alanine aminotransferase, *AST* aspartate aminotransferase

### Ethics

Ethical approvals have been confirmed from the Institutional Review Board at Guangdong Provincial Hospital of Chinese Medicine (BF2018–100-01), The First Affiliated Hospital of Guangzhou University of Chinese Medicine (ZYYECK【2019】006) and The Third Hospital of Nanchang (2018–011). The trial was registered in the Chinese Clinical Trials Registry with ID: ChiCTR1800020107. Patients will receive a detailed information sheet and complete written consent forms.

The trial is managed by the School of Chinese Medicine, Hong Kong University and data will be supervised by the Data and Safety Monitoring Board (DSMB), which is an independent group of experts that advises funding agency and study investigators. DSMB members include three experts from different fields (western medical sciences, Chinese medicine and statistics). The DSMB is for quality control of this research data and ensures the integrity of the study. The protocol compliance, safety, and on-schedule study progress are also monitored by the DSMB. An auditing trial will be conducted every 3 months and the process will be independent from investigators. Study documents (soft and hard copies) will be retained in a secure location for 5 years after trial completion.

### Subjects

A total of 214 eligible patients will be recruited at different clinical centers. Inclusion criteria include: (1) women with stage-I or -II breast cancer aged between 18 and 75 years; (2) receiving first chemotherapy; (3) planning to receive chemotherapeutic agents containing taxanes or/and anthracyclines; (4) the chemotherapy regimen will last for at least six cycles with every 3 weeks a cycle; (5) adverse events assessed using WHO toxicity classification criteria < grade II; and (6) a life expectancy of at least 6 months. Exclusion criteria are: (1) patients with a medical history of hair transplantation; (2) patients suffering from psoriasis or severe scalp infection; (3) hair loss induced by alopecia areata, alopecia totalis or scalp injury, etc.; (4) pregnancy, lactation or potential pregnancy; (5) allergy to some specific foods, like black soybean; (6) severe cardiac, hepatic, renal, pulmonary and hematic lesions or other diseases which will affect their survival; (7) those who have any severe mental or behavioral disorders who cannot be fully informed; (8) suspected of or with a history of alcohol and/or drug abuse; (9) cannot understand or fill in questionnaires because of cognitive disorders or a low level of literacy; and (10) a variety of factors affecting drug taking and absorption, such as the inability to swallow, chronic diarrhea, intestinal obstruction, etc. Eligible patients will be invited to participate in this study after obtaining their written consent form. All participants will be closely monitored in the study.

### Estimation of sample size

The primary outcome in this study is the time point of occurrence of chemotherapy-induced hair loss reaching grade II measured by the WHO Toxicity Grading Scale for Determining the Severity of Adverse Events. Our previous results showed that YH0618 could cause the incidence of hair loss grade < II to reach 50% for patients who have completed chemotherapy, and the difference between the incidence of hair loss grade < II in YH0618 group and the control group was 15%. Thus, the difference in proportion between the two groups will be measured by a Z test. To achieve a two-sided, type-I error alpha = 0.05 and power: (1 − beta) = 80%, the minimal number of subject need in each group is 85. We estimated a 20% attrition rate at the end of follow-up; hence, a sample size of at least 107 in each group (214 in total) is planned for this study.

### Randomization and blinding

Each subject will obtain an unique number after completing written consent. A computer-blocked random number sequence with a block size of four will be generated centrally by a statistician not involving in this study. As YH0618 granule and placebo show the same appearance, a double-blind model will be adopted. Therefore, the randomization sequence and different groups will be kept hidden from subjects, practitioners, data collectors and statisticians.

### Intervention and control condition

Prior to intervention, baseline data will be collected including demographics, medical characteristics, assessment of chemotherapy-induced hair loss, facial color, thumbnail color, grading of thumbnails ridging, quality of life, blood routine test results, and hepatic and renal function. After that, subjects assigned to group A will receive YH0618 granules three times a day (6 g each time), 6 days a week, mixed with 300 ml warm water from the first to the fourth chemotherapy cycle. Subjects in group B will receive the placebo granule in the same manner. Then, all the subjects will be followed up for 1 month after chemotherapy. All specific methods, such as scalp cooling, used for reducing hair loss will be prohibited during the clinical trial. Both YH0618 granules and placebo are produced by Guangzhou Kanghe Pharmaceutical Co., Ltd., which meets national standards.

### Outcome evaluation

#### Primary outcome

The primary outcome is the time point of occurrence of hair loss reaching grade II as assessed by the WHO Toxicity Grading Scale, and objective indices of hair quality and hair-follicle growth recorded by a hair and scalp detector (CBS-603, CBS, Taiwan) before the fifth chemotherapy cycle.

### Grading of chemotherapy-induced hair loss

The WHO Toxicity Grading Scale is commonly used to monitor and rate the severity of anticancer drug-induced toxicity [19, 20]. The grading criteria for hair loss is shown in Table 2. Alopecia assessments will be conducted by a clinician blinded to treatment assignment, and by the participant.

**Table 2:** World Health Organization (WHO) grading criteria for anti-drug induced hair loss

Grade 0	No hair loss
Grade I	Minor hair loss
Grade II	Moderate hair loss/alopecia areata
Grade III	Complete hair loss with regrowth
Grade IV	Non-regrowth hair loss

#### Objective measurement of hair loss

In order to objectively evaluate the hair quality and hair-follicle growth, a hair and scalp detector (CBS-603) will be used. The detector obtained patents in the United States, German, Japan, China, and China Taiwan, and many international authentications from Conformité Européenne (CE), Federal Communications Commission (FCC), and Restriction of Hazardous Substances (RHoS). The detector is composed of a 10X-200X Hair and Scalp Camera and software. The whole top of the head, a wide range of hair loss and the condition of hair follicles could be clearly filmed at 10X, 50X and 200X, respectively. The software has a function of testing through which hair testing and analysis can be conducted. In this study, identification and classification of the level of hair loss, hair diameter and quality will be analyzed.

### Secondary outcomes

Secondary outcomes include changes of facial color and thumbnail color, grading of thumbnail ridging, assessment of quality life, fatigue level, routine blood test results, hepatic and renal function, and certain medical indicators which can reflect kidney deficiency in Chinese Medicine.

#### Facial color and thumbnail color

The assessment of facial and thumbnail color is performed using the L*a*b system, which is the same as for the clinical trial that we conducted previously [13]. In the fixed surroundings, the skin color of the forehead, right and left cheeks, and jaw, and the thumbnail color will be recorded by the hair and scalp detector at 50X.

#### Grading of thumbnail ridging

The grading of left and right thumbnail ridging will be measured by the National Cancer Institute Common Terminology Criteria for Adverse Events (NCI CTCAE). The definition of nail ridging is a disorder characterized by vertical or horizontal ridges on the nails. The grading criteria for nail ridging is shown in Table 3.

**Table 3:** Criteria for assessing chemotherapy-induced nail ridging

Grade 1	Asymptomatic; clinical or diagnostic observations only; intervention not indicated
Grade 2	Distortion of nail shape; associated psychosocial impact

Definition of nail ridging: a disorder characterized by vertical or horizontal ridges on the nails

#### Quality of life measurement

Quality of life has been regarded as an important index to measure and monitor cancer patients’ treatment outcomes [21]. The Chinese version of the Functional Assessment of Cancer Therapy-Breast Cancer (FACT-B) with good reliability and validity will be used to measure breast-cancer-specific quality of life [22]. The tool includes 37 items scored on a 5-point Likert scale, ranging from 0 to 4 with higher scores indicating better quality of life [23, 24]. The items are classified into five subscales: Physical Well-Being, Social/Family Well-Being, Emotional Well-Being and Functional Well-Being, which constitute the FACT-General, and the additional concern for breast cancer, which is called the Breast Cancer Subscale. A total score is calculated by summing all subscale scores.

#### Fatigue measurement

Fatigue will be measured by the Chinese version of FACIT-Fatigue version 4, a 13-item FACIT Fatigue Scale, which could be used for patients with any tumor type [25]. Each item scored on a 5-point Likert self-report scale, ranging from 0 to 4. A total score is obtained by summing all item scores and a high score indicates less fatigue.

#### Clinical objective examination

Routine blood tests and assessment of liver and kidney function are the same as in our previous trial [13]. Based on the evaluation standard of kidney deficiency in Chinese Medicine, kidney deficiency will be divided into deficiency of kidney *qi*, deficiency of kidney *yang* and deficiency of kidney *yin*. Modern studies also found that kidney deficiency syndrome has a modern pathophysiological basis, clinically manifested as changes in the relevant medical indicators such as the adrenal axis, thyroid axis, gonadal axis, renin-angiotensin system, immune energy, liver and kidney function and hematopoietic function [26]. So, in this study, immune indices include immunoglobulin M (IgM), alexin C3, helper T cells CD4+, CD8+ T cells and certain metabolic indices of microelements such as Mg^2+^, Cu^2+^, Zn^2+^, and Fe^3+^.

All participants will be assessed within 3 days before every chemotherapy cycle from the first to the fifth cycle. Then, all the subjects will be followed up and the final assessment will be conducted at 1 month after the last chemotherapy cycle. A professional research assistant will assign YH0618 granules or placebo, and notify the subjects of dosage and time. Quality and compliance to the intervention will be achieved by checking attendance records and the self-record diary kept by each participant.

### Adverse events

Adverse events will be recorded spontaneously through self-reports by participants or asking the participants the open-ended question “How are you feeling?” via phone or face to face. Any questions concerning adverse events will be reported regardless of whether they were deemed to be related to the treatment be assessors and will be sent to the Institutional Review Board of every clinical center.

### Statistical analysis

All analyses will be performed based on intention-to-treat principles, any missing data in the follow-up visits will be imputed using multiple imputation. Descriptive analyses as means and standard deviations (SDs) will be used to describe the demographics and clinical characteristics of the participants. The primary efficacy analysis compared the hair-loss grading between YH0618 granule and control before the fifth chemotherapy using Fisher’s exact test. The changes of hair diameter between the two groups after four cycles of chemotherapy will be compared by an independent samples *t* test. A multivariable logistic regression model will be used to explore the treatment effect. Potential confounding variables will be identified as those that differ among treatment groups at baseline and are significantly associated with outcomes. Changes from baseline to the final assessment in quality of life assessed by the FACT-B and objective indicators in the blood will be compared using Wilcoxon rank sum tests. Unless otherwise specified, two-sided statistical tests will be used and the significance level will be set at *p* < 0.05.

In this trial, an interim analysis will be performed when approximately two thirds of the planned observations are enrolled. The results are analyzed by the statistician and only DSMB members have access to the results to test for futility, safety and efficacy of the trial. A predefined stopping rule will be applied to the data to determine whether it is futile to continue enrollment.

## Discussion

Chemotherapy-induced hair loss occurs usually due to the high mitotic rate of hair follicles instead of a non-androgenic mechanism, and can manifest as alopecia totalis, telogen effluvium, or, less often, as alopecia areata [27]. Severe hair loss occurs most often with drugs such as doxorubicin, daunorubicin, docetaxel, paclitaxel, cyclophosphamide and etoposide, which are common chemotherapy agents used for breast cancer patients. Even some standard chemotherapy regimens can induce permanent thinning or hair loss. Although scalp-cooling is a method approved by the FDA for preventing both permanent and temporary hair loss, concerns about this method have been raised [6, 28]. Therefore, this is the first strict randomized, double-blind, multi-center controlled trial to evaluate the effect of a medicinal and edible compound prescription on chemotherapy-induced hair loss. The proposed study may provide direct and convincing evidence to support YH0618 as an adjuvant treatment for reducing chemotherapy-induced toxicity, which could be introduced into clinical settings. Our achievements will provide a safe and effective way for reducing chemotherapy-induced hair loss and improving patients’ quality of life.

## Trial status

The protocol version 1. Recruitment will start in June 2019 and the trial is expected to be completed in December 2020.

## Supplementary information

**Additional file 1.** Standard Protocol Items: Recommendations for Interventional Trials (SPIRIT) Checklist.

## Data Availability

Not applicable

## Abbreviations

ALT: Alanine aminotransferase
AST: Aspartate transaminase
BUN: Blood urea nitrogen
CE: Conformité Européenne
DSMB: Data and Safety Monitoring Board
FACT-B: Functional Assessment of Cancer Therapy-Breast Cancer
FCC: Federal Communications Commission
FDA: Food and Drug Administration
IgM: Immunoglobulin M
RHoS: Restriction of Hazardous Substances
UA: Uric acid
WHO: World Health Organization
